# The Adaptive Immune Response against *Bunyavirales*

**DOI:** 10.3390/v16030483

**Published:** 2024-03-21

**Authors:** Reem Alatrash, Bobby Brooke Herrera

**Affiliations:** 1 Rutgers Global Health Institute, Rutgers University, New Brunswick, NJ 08901, USA; 2 Department of Medicine, Division of Allergy, Immunology, and Infectious Diseases and Child Health Institute of New Jersey, Rutgers Robert Wood Johnson Medical School, New Brunswick, NJ 08901, USA

**Keywords:** T cells, antibodies, *Bunyavirales*, *Peribunyaviridae*, *Phenuiviridae*, *Hantaviridae*, *Nairoviridae*, *Arenaviridae*, bunyaviruses

## Abstract

The *Bunyavirales* order includes at least fourteen families with diverse but related viruses, which are transmitted to vertebrate hosts by arthropod or rodent vectors. These viruses are responsible for an increasing number of outbreaks worldwide and represent a threat to public health. Infection in humans can be asymptomatic, or it may present with a range of conditions from a mild, febrile illness to severe hemorrhagic syndromes and/or neurological complications. There is a need to develop safe and effective vaccines, a process requiring better understanding of the adaptive immune responses involved during infection. This review highlights the most recent findings regarding T cell and antibody responses to the five *Bunyavirales* families with known human pathogens (*Peribunyaviridae*, *Phenuiviridae*, *Hantaviridae*, *Nairoviridae*, and *Arenaviridae*). Future studies that define and characterize mechanistic correlates of protection against *Bunyavirales* infections or disease will help inform the development of effective vaccines.

## 1. Introduction

The *Bunyavirales* order, as delineated by the International Committee on Taxonomy of Viruses (ICTV), encompasses hundreds of viruses, colloquially known as “bunyaviruses”, distributed across at least fourteen viral families (*Arenaviridae*, *Cruliviridae*, *Discoviridae*, *Fimoviridae*, *Hantaviridae*, *Leishbuviridae*, *Mypoviridae*, *Nairoviridae*, *Peribunyaviridae*, *Phasmaviridae*, *Phenuiviridae*, *Tospoviridae*, *Tulasviridae*, and *Wupedeviridae*) [1,2,3]. Apart from hantaviruses and arenaviruses, which are primarily transmitted by rodents, most viruses of the *Bunyavirales* order rely on arthropod vectors like mosquitoes, ticks, and sandflies for transmission [4,5]. The proliferation of these vectors, influenced significantly by climate change, has expanded the geographical reach of *Bunyavirales*, including countries across the Americas, Europe, Asia, the Middle East, and Africa [6,7].

While the majority of viruses within the *Bunyavirales* order are not inherently pathogenic to humans, five families (*Peribunyaviridae*, *Phenuiviridae*, *Hantaviridae*, *Nairoviridae*, and *Arenaviridae*) include viruses responsible for human and other vertebrate infections. Human infection may manifest as a mild, febrile illness with the potential to progress to fatal hepatitis, hemorrhagic fever, or encephalitis [8]. To date, specific vaccines or antivirals for the prevention or treatment of *Bunyavirales* infections are not approved. Given the escalating risk of human exposure to these emerging and re-emerging viruses, there is a need to develop effective vaccines, crucial not only for infection prevention but also to curb the emergence of severe disease.

In this review, we provide a comprehensive examination of adaptive immune responses against the five *Bunyavirales* families with known human pathogens (*Peribunyaviridae*, *Phenuiviridae*, *Hantaviridae*, *Nairoviridae*, and *Arenaviridae*), identify current gaps in our understanding of protective mechanisms against these viruses, and suggest future research priorities to address the existing unknowns in the field.

## 2. *Bunyavirales* Structure and Life Cycle

**Figure 1:**
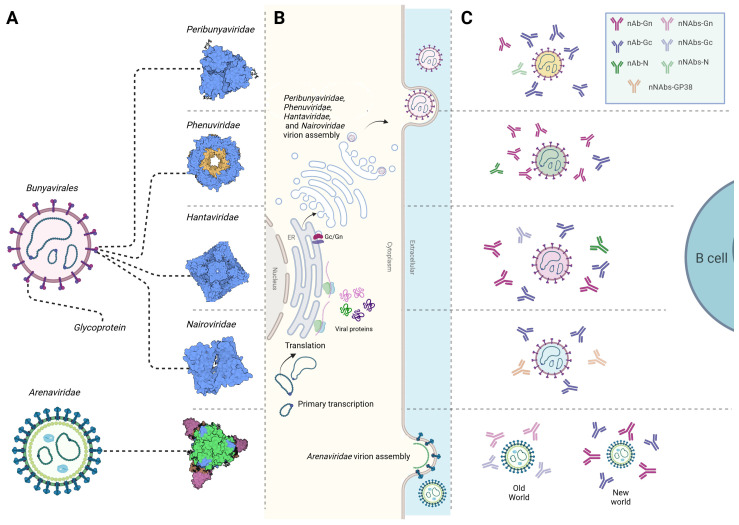
*Bunyavirales* virions and envelopes, infectious cycle, and antibody response. (**A**) Schematic representation of *Peribunyaviridae*, *Phenuinviridae*, *Hantaviridae*, *Nairoviridae*, and *Arenaviridae* envelope glycoproteins that enable virus entry into host cells. (**B**) Schematic representation of *Bunyavirales* infectious cycles, depicting viral RNA in the host cytoplasm initiating replication of infectious virus components. Newly synthesized glycoproteins of viruses within *Peribunyaviridae*, *Phenuinviridae*, *Hantaviridae*, and *Nairoviridae* form oligomers within the endoplasmic reticulum membrane and traffic to the Golgi apparatus for virion assembly and the subsequent budding of infectious particles. In contrast, *Arenaviridae* virions assemble at the cell membrane. (**C**) Schematic representation of neutralizing and non-neutralizing antibodies against *Bunyavirales*. Antibodies are known to target specific *Bunyavirales* proteins, typically Gc, Gn, and N, with each virus family having its own distinct major targets and neutralizing capabilities due to the viruses’ intricate nature. For the *Peribunyaviridae* family, nAbs mainly target Gc, whereas antibodies against N are generally less common and non-neutralizing. For *Phenuinviridae*, Gn is the main target for nAbs, with N and Gc proteins showing relatively less neutralization potential. For *Hantaviridae*, the most effective nAbs are against Gn and Gc. For Nairoviridae, Gc has been demonstrated as the primary target for the host nAbs, while other nNAbs directed at GP38 have been found to confer protection in rodent models. For *Arenaviridae*, different immunological patterns have been noted in response to Old and New World viruses. Typically, New World arenaviruses induce strong nAbs, while Old World arenaviruses often evade these responses. Glycoprotein structures were retrieved from PDB (*Peribunyaviridae* represented by La Crosse virus: 6H3W; *Phenuiviridae* represented by Rift Valley fever virus: 6F9F; Hantaviridae represented by Andes virus: 6ZJM; *Nairovoridae* represented by Crimean–Congo hemorrhagic fever virus: 8DC5; and, *Arenaviridae* represented by Lassa virus: 8EJH). nAb: neutralizing antibodies, nNAbs: non-neutralizing antibodies.

Viruses within the *Bunyavirales* order contain enveloped, segmented single-stranded ribonucleic acid (RNA) genomes of negative-sense or ambisense polarity [9]. Except for the *Arenaviridae* family [10], viruses within *Peribunyaviridae*, *Phenuiviridae*, *Hantaviridae*, and *Nairoviridae* families share a conserved genetic organization comprising three segments, classified by size as small (S), medium (M), and large (L) (Figure 1A). Each segment serves as a template for positive-sense antigenome replication and mRNA transcription. The S segment encodes the nucleocapsid protein (N) and nonstructural protein s (NSs), which has been shown to modulate the host cell antiviral response through innate immune pathways [11]. The M segment encodes a glycosylated polyprotein precursor (GPC) that undergoes cleavage by host cell proteases, resulting in the production of the envelope spike proteins Gn and Gc [12,13]. In certain virus species, nonstructural protein m (NSm) has been shown to play a role in viral assembly and regulation of apoptosis [12,13,14]. The L segment encodes the L protein, an RNA-dependent RNA polymerase (RdRp) responsible for transcription and replication of the S, M, and L segments [15].

Viruses within the *Bunyavirales* order exhibit diverse envelope glycoproteins that enable viral entry into host cells through surface receptors, many of which remain unidentified (Figure 1A). Nevertheless, studies have identified dendritic cell-specific intercellular adhesion molecule-3-grabbing non-integrin (DC-SIGN) and liver/lymph node-specific intercellular adhesion molecule-3-grabbing non-integrin (L-SIGN) as receptors for viral entry for several viruses within the *Arenaviridae*, *Nairoviridea* and *Phenuiviridae* families [16,17,18]. While DC-SIGN and L-SIGN have been implicated in *Bunyavirales* entry, it is important to note that they likely serve as non-specific receptors, given that they are found on the surface of dermal dendritic cells where vector-borne viruses are typically transmitted [19]. *Bunyavirales* enter host cells via clathrin-mediated, caveolin-mediated, or independent endocytosis [20,21]. After cell entry, virions fuse with endosomes, and due to exposure to a low pH, conformational changes are triggered in the glycoproteins. This event leads to virion uncoating and the presentation of viral RNA in the host cytoplasm, initiating viral replication. RdRp from the infectious particle facilitates genome replication, followed by synthesis of all infectious virus components [22]. Newly synthesized glycoproteins rapidly form oligomers within the endoplasmic reticulum (ER) membrane, subsequently trafficking to the Golgi apparatus for virion assembly (Figure 1B). In the lumen of the Golgi, the newly assembled genome segments interact with the cytoplasmic tail of Gn and are packaged into viral particles [23]. Subsequently, the progeny viruses bud into the secretory vesicles and traffic towards the plasma membrane, where they are released into the extracellular space, although the pathway by which virions are secreted remains unclear [23]. In contrast, arenaviruses and some hantaviruses have been shown to assemble and bud at the cell surface or plasma membrane, distinguishing them from other viruses within the *Bunyavirales* order [24,25,26]. Specific details regarding *Bunyavirales* structures and life cycles have been extensively reviewed elsewhere [27].

## 3. T Cell Responses against *Bunyavirales*

### 3.1. Peribunyaviridae

The *Peribunyaviridae* family currently encompasses 7 genera and 219 virus species. Among these, the *Orthobunyavirus* genus, including the California (CSG), Simbu (SSG), and Bunyamwera serogroups, are the most extensively studied for their ability to cause human infection [28,29]. This review specifically focuses on the adaptive immune response to orthobunyaviruses.

The characterization of immunodominant T cell epitopes is lacking for most orthobunyaviruses. Previous studies have primarily observed T cell responses against specific protein subunits, mainly GPC and/or N [30,31]. Immunoinformatic studies have identified and predicted virus-specific CD4+ and CD8+ T cell epitopes within GPC and N for the Jamestown Canyon (JCV), Oropouche Virus (OROV), and Bunyamwera virus (BUNV), showing a high affinity with human major histocompatibility complex class I (MHC-I) and MHC-II (Table 1) [30,31,32,33]. While these studies suggest the potential development and use of multi-epitope vaccines, future in vivo studies are required to validate immunogenicity, efficacy, and protection. Notably, the immunogenicity of glycoproteins and N has been confirmed in mice lacking interferon alpha/beta receptors (IFNAR^−/−^) for the La Crosse virus (LACV) and Schmallenberg virus (SBV) (Table 1) [34,35]. Ex vivo studies confirming T cell responses to entire proteomes have not been conducted for any virus in this family.

In the case of LACV, DNA vaccination in IFNAR^−/−^ mice with LACV Gn/Gc induced complete protection mediated by CD4+ T cells, while N DNA vaccination provided partial protection [35]. Similar findings were observed for SBV vaccination, where DNA encoding the Gc ectodomain induced CD8+ T cell-mediated protection [34]. Additionally, subunit- or DNA-based N vaccines have both been shown to reduce clinical signs and significantly decrease viremia upon SBV challenge, eliciting CD8+ T cell-mediated responses [34,36].

While most studies on T cell responses to orthobunyaviruses have been conducted using animal models, human T cell responses remain understudied. Notably, LACV’s clinical importance has led to comprehensive studies on cellular responses, especially considering its status as the leading cause of pediatric viral encephalitis in the United States [64]. Children exhibit higher susceptibility to LACV infection, while adults typically experience asymptomatic or mild infections. This age-related susceptibility can be recapitulated in murine models, providing opportunities to study innate and adaptive immune responses against LACV and other related orthobunyaviruses.

In wild-type C57BL/6 mice, both CD4+ and CD8+ T cells (and B cells) play a role in LACV infection [65]. Although these cell types did not impact neurological disease in weanling mice (3–4 weeks old), they were crucial for protecting adult mice (6–8 weeks old) from LACV pathogenesis [65]. Natural killer (NK) cells did not appear to have a major role in protection against LACV, as their depletion in adult mice did not impact pathogenesis [65]. These findings underscore the importance of the adaptive immune response in preventing LACV neurological disease, beyond the innate immune response alone.

Given the limited information on T cell responses and immunodominant epitopes, progress in vaccine development for viruses within the *Peribunyaviridae* family is hampered. The characterization of adaptive immune responses to natural infection, along with the in vivo validation of computationally predicted vaccine peptides, is essential to overcome this gap.

### 3.2. Phenuiviridae

The *Phenuiviridae* family currently encompasses 22 genera and 151 virus species, demonstrating a broad host range that includes humans, animals, plants, and fungi [66]. In 2018, the World Health Organization identified the Rift Valley fever virus (RVFV) and severe fever with thrombocytopenia syndrome virus (SFTSV) as emerging threats, underscoring the urgency for accelerated research and development efforts [67]. RVFV, transmitted by mosquitoes, is prevalent across Africa and the Middle East. Although typically inducing mild, self-limiting disease, severe complications such as hepatitis, encephalitis, or death can occur [68]. The impact and economic toll of RVFV on local domestic livestock, including abortion storms, highlights the significant risks it poses to global food security and public health [69]. SFTSV, transmitted by ticks, causes a highly fatal condition marked by hemorrhagic symptoms [70]. Toscana virus (TOSV), another re-emerging member of this family, ranks among the top etiological agents of aseptic meningitis, and is transmitted by sand flies [71].

Efforts have been made to identify RVFV, SFTSV, and TOSV epitopes targeted by T cells. Using immunoinformatic approaches, TOSV studies identified T cell epitopes within Gn, Gn, and N, leaving the rest of the viral proteome with unknown immunogenicity [37]. In contrast, the entire proteomes of RVFV and SFTSV were analyzed for immunogenicity, revealing immunodominant T cell epitopes within glycoproteins and N but also nonstructural proteins (Table 1) [38,39].

In humans vaccinated with formalin-inactivated RVFV, CD4+ and CD8+ T cell targets within Gn, Gc, and N were confirmed in ex vivo assays (Table 1) [40]. Another research group defined two HLA-A-2-directed RVFV N epitopes using N-transduced dendritic cells (DCs) to prime CD8+ T cells from HLA-A-2 donors [41]. In vivo assays of T cell responses in mice vaccinated with attenuated RVFV strains also demonstrated that two epitopes within N were robustly targeted (Table 1) [42]. For SFTSV, immunoinformatic screening suggested peptides within RdRp and glycoproteins, leading to the in silico evaluation of a multi-epitope vaccine (Table 1) [38]. The same approach predicted CD4+ T cell responses against epitopes within the TOSV N and glycoproteins as being highly immunogenic (Table 1) [37]. However, the in vivo evaluation of epitope-specific T cells from humans vaccinated or infected with SFTSV or TOSV has not been performed.

The immunogenicity of Gn, Gc, and N for RVFV, SFTSV and TOSV has been confirmed with vaccine studies in animal models, emphasizing a protective role for virus-specific CD4+ and CD8+ T cells [72,73,74,75,76,77,78,79,80]. Gn immunization in goats using a recombinant Capripoxvirus vaccine induced protection against RVFV challenge, mediated through a CD4+ T cell response [72]. BALB/c mice vaccinated with a single dose of a DNA vaccine encoding the RVFV Gn/Gc showed no viremia or clinical disease, with glycoprotein-specific CD8+ T cell responses, while N-based vaccination conferred only partial protection [73]. Notably, this vaccine regimen failed to protect IFNAR^−/−^ mice from RVFV lethal infection, suggesting the involvement of innate immunity in protection [73]. For SFTSV, vaccine studies involving ferrets and IFNAR^−/−^ mice revealed that a DNA plasmid encoding Gn/Gc induced protection primarily through antigen-specific T cell responses [75,76,77,78,79]. This effect occurred in the absence of detectable antibodies against surface glycoproteins in immunized mice [76]. The same antigens, when produced via mRNA vaccination, induced a balanced Th1/Th2 response in mice [77,78]. Similarly, BALB/c mice were fully protected from a lethal dose of TOSV when vaccinated with recombinant Gc and N (but not when vaccinated with single antigens), demonstrating a potent CD8+ T cell response associated with significant IFN-γ expression [80].

Furthermore, studies in C57BL/6 mice showed that CD4+ T cells, largely Type 1 T helper cells (Th1)/T follicular helper cells (Tfh) subtypes, play a protective role, with the T-box transcription factor TBX21 (T-bet), Cluster of Differentiation 40 (CD40), Cluster of Differentiation 40 Ligand (CD40L), and MCH II pathways crucial in mediating defense against RVFV encephalitis [42]. In a separate study using immunocompetent mice, infection with an attenuated RVFV strain induced an expansion of NK cells, monocytes, and both CD4+ and CD8+ T cells [81]. Depleting C57Bl/6 mice of CD4+ and CD8+ T cells increased the frequency of encephalitis, supporting that these cell types contribute to the prevention of disease [81,82]. It is worth noting that adaptive immune responses against RVFV, due to its rapid progression and high lethality in rodent models, have mostly been explored using attenuated strains or recombinant viral proteins [42,81,83,84,85]. In contrast, T cell responses to SFTSV have been well studied in human patients. Non-surviving patients exhibit decreased cell counts, including CD3+, CD4+, and CD8+ T cells, suggesting immune dysfunction in SFTSV disease progression [86,87]. CD4+ T cell deficiency and Th1/Th2 imbalance correlate with increased viral load, serum enzymes, cytokines, and disease severity [88,89,90,91]. Surviving patients have an increased expression of activation markers in T cells [92]. Specifically, CD8+ T cells exhibit a proliferative activated phenotype demonstrated by an increased expression of CD69 and CD25, secreting a higher level of IFN-γ and granzyme B with enhanced antiviral responses, further supporting that cellular responses play a protective role against infection [93].

Studies on human T cell responses against TOSV are lacking, as well as on other clinically important viruses within the *Phenuiviridae* family, including the Heartland virus (HRTV), Arumowot virus (AMTV), Uukuniemi virus (UUKV), Guertu virus (GTV), Punta Toro virus (PTV), and sandfly fever Sicilian virus (SFSV). Future studies are needed to better understand cellular immunity against these globally relevant pathogens, aiming to identify correlates of protective immunity that will aid the development of vaccines.

### 3.3. Hantaviridae

The *Hantaviridae* family encompasses 7 genera and 54 species, responsible for diverse human diseases. Old World hantaviruses in Asia and Europe cause hemorrhagic fever with renal syndrome (HFRS), while New World hantaviruses in North and South America induce hantavirus cardiopulmonary syndrome (HCPS) [94]. Hantaviruses have evolved multiple immune evasion strategies to establish long-term infections in their natural hosts without causing noticeable illness, in order to ensure their survival and facilitate transmission to other hosts, including humans [95]. Hantaviruses can downregulate viral antigen expression and interfere with host antiviral responses by modulating cellular signaling pathways, as well as suppressing the immune response by inducing regulatory T cells, enabling the virus to persist [96]. The role of the adaptive immune response in either protection or pathogenesis remains a topic of ongoing investigation [97,98].

Immunoinformatic studies have aimed to identify immunodominant T cell epitopes within hantavirus proteins, with the majority revealing epitopes in N followed by glycoproteins (Table 1) [43,49,99]. When T cell responses against entire proteomes were analyzed for orthohantaviruses, epitopes within glycoproteins, N, and RdRp and other non-structural proteins were predicted to have high immunogenicity (Table 1) [43]. In 1999, the first demonstration of human T cell responses to the Hantan virus (HTNV) suggested that the CD8+ T cells elicited upon infection are limited to N, recognizing two immunodominant epitopes [44]. Advances in the field in later years revealed an expanded panel of immunodominant epitopes within the HTNV N in HFRS patients (Table 1) [45,46,100]. Further detailed characterization suggested HTNV N epitopes restricted by various human leukocyte antigens (HLAs), conserved in both HTNV and Sin Nombre virus (SNV) (Table 1) [47,48]. Moreover, the cross-reactivity of N-specific CD8+ T cells against several hantaviruses has been reported in human studies [44,46,101]. Additionally, multiple observations of Gn- and Gc-specific T cell responses have been reported in patients infected with HTNV and the Andes virus (ANDV) (Table 1) [49,50]. ANDV epitopes located within the Gn carboxyl-terminus were immunodominant, as compared to those from within N and Gc in HCPS patients, and CD8+ T cells targeting ANDV Gn acquire a long-lasting effector phenotype [50]. CD8+ T cells from patients infected with the Puumala virus (PUUV) also exhibit strong responses against a recombinant vaccinia virus expressing N and the second half of Gn [51]. Notably, virus-specific CD8+ T cell responses during HFRS play a crucial role in HTNV clearance, being efficient releasers of cytotoxic mediators, adopting a memory effector phenotype and its recruitment at an early stage of HFRS [100,102,103,104]. Similarly, an increase in both CD4+ and CD8+ T cells across disease stages correlates with delayed viral clearance in HCPS patients [105,106].

The involvement of CD8+ T cells in hantavirus infection is not fully elucidated. Findings in human patients demonstrated a proportional increase in circulating HTNV-infected CD8+ T cells and disease severity [107]. A recent study in HCPS patients observed an increase in both CD4+ and CD8+ T cells across disease stages, correlating with delayed viral clearance, while in HFRS, the frequency of HTNV-specific effector CD8+ T cells is higher during mild stages compared to the acute phase [105,106]. While CD4+ T cell responses have received less attention, studies suggest a mixed Th1/Th2 profile, based on cytokine profiles in HTNV-infected human sera [108,109]. However, there is no clear correlation between effector CD4+ T cells and clinical outcomes.

Insights into protective hantavirus-specific T cell responses have also been obtained from antigen immunization using animal models. BALB/c mice vaccinated with *E. coli*-expressed PUUV N developed proliferative Th cells that secreted immune modulators [110]. The HTNV N- and glycoprotein-derived immunodominant epitopes previously identified using in silico methods were used to immunize HLA-A2.1/K(b) transgenic mice, both inducing protective T cell responses [49,99,111] Notably, immunization with a multi-epitope HTNV vaccine containing subunits of both N and glycoprotein produced stronger T cell responses, compared to single immunization with either epitope in both human cells and transgenic mice [112,113].

N protein is relatively conserved and highly immunogenic among hantaviruses [110,114,115,116]. Given this observation, a study demonstrated cross-protective immune responses against PUUV, Topografov virus (TOPV), ANDV, and Dobrava virus (DOBV) by immunizing bank voles with recombinant N (rN) from different hantaviruses [117]. When rN-immunized mice were challenged against PUUV, cellular responses were more instrumental than the humoral response in this cross-protective immunity [117]. Based on this cross-reactivity study and all the previously mentioned findings, a universal T cell-based vaccine targeting multiple viruses might be achievable and promising in the case of hantaviruses. Additionally, given that multiple immunodominant epitopes within N, Gn, and Gc have been identified in different studies, an unbiased screening of T cell responses against conserved regions of the hantavirus proteome may enable a narrowing down of immunodominant targets that could be useful for cross-protective vaccine development.

### 3.4. Nairoviridae

The *Nairoviridae* family currently encompasses 3 genera and 58 virus species. These viruses are maintained in arthropods and transmitted primarily by ticks to mammals, birds, and bats. Among them, the most significant human pathogen is the Crimean–Congo hemorrhagic fever virus (CCHFV), prevalent in Asia, Africa, and Southern and Eastern Europe [118]. The Nairobi sheep disease virus (NSDV) is also noteworthy within this family due to its veterinary impact, causing highly lethal disease in small ruminants in Africa and India [119].

Research efforts to better understand adaptive immune responses against CCHFV have addressed notable gaps [120]. Using immunoinformatic approaches, several studies identified CD4+ and CD8+ T cell-specific epitopes within CCHFV GPC, N, and RdRp proteins (Table 1) [52,53,121]. In silico analysis further pinpointed six regions of the CCHFV glycoprotein with high antigenic potential [52]. The epitope “DCSSTPPDR” in RdRp was also identified as particularly immunogenic (Table 1) [54]. Furthermore, CCHFV survivors demonstrated strong IFN-γ responses against the NSm region of the GP38 protein in ex vivo assays (Table 1) [55]. Another study of CCHFV survivors identified cellular responses against N, indicating a preference for non-Gn/Gc epitopes [56]. Confirming these human findings, immunodominant epitopes were also identified in the N-terminus of Gc followed by NSm as the primary CD8+ T cell targets in CCHFV-infected mice [57].

Vaccine studies have also supported a role for protective T cell responses against CCHFV challenge [122,123]. Mice vaccinated with DNA encoding the CCHFV GPC protected against disease, mediated primarily by CD8+ T cells [122]. However, in a separate study, signal transducer and activator of transcription 1 knockout (STAT1^−/−^) mice immunized with the Gn and Gc ectodomains failed to be protected against disease upon CCHFV challenge, even with detectable serum neutralizing antibodies (nAbs) [124]. In a separate study, IFNAR^−/−^ mice immunized with nucleoside-modified mRNA-lipid nanoparticles encoding CCHFV glycoproteins or N demonstrated strong, protective cellular immune responses [123]. An adoptive transfer of serum Abs and T cells from mice immunized with a modified vaccinia Ankara virus vector expressing the CCHFV glycoprotein protected recipient mice against lethal challenge [125]. Depletion of either CD4+ or CD8+ T cells significantly increased mortality in infected mice, underscoring the essential role for these cell types in protection against severe disease [126]. Finally, recent findings have also highlighted a crucial role for CD8+ T cells in efficiently controlling acute infection in wild-type mice, rapidly acquiring CCHFV-specific antiviral effector functions, including the production of antiviral cytokines [57].

While early studies in CCHFV patients suggest that cellular immunity enhances survival during acute infection [56,127], the exact mechanisms by which T cells contribute to survival remain to be investigated. Adaptive immune responses to other nairoviruses, especially NSV, are also underexplored. The Hazara virus (HAZV), closely related to CCHFV, has served as a biosafety level 2 (BSL-2) surrogate model for CCHFV research, facilitating research without the requirement and constraints of a high-containment BSL-4 environment. Studies on HAZV have helped reveal important insights into CCHFV immunopathogenesis; however, ex vivo and in vivo studies exploring T cell responses against HAZV require further investigation [128,129]. Further understanding the mechanisms of viral clearance mediated by T cells will be important for designing effective vaccines against CCHFV and other nairoviruses.

### 3.5. Arenaviridae

The *Arenaviridae* family currently encompasses 5 genera and 74 virus species with the capability of causing infections in diverse hosts. Mammarenaviruses, which include pathogens typically not infecting mammals beyond their primary reservoir hosts, post a threat to humans through direct contact with infected rodents, their droppings, or urine, the ingestion of contaminated food, or the inhalation of aerosolized droplets from contaminated rodent excreta, secreta, or body parts [130]. Human diseases caused by mammarenaviruses include Lassa fever (LF), caused by the Lassa virus (LASV) in Western Africa. The Lujo virus (LUJV) has also recently caused a small but severe outbreak in Southern Africa [131]. Other mammarenaviruses, including the Junin (JUNV), Machupo (MACV), Guanarito (GTOV), Sabia (SBAV), and Chapare (CHAPV) viruses, cause human disease most often associated with hemorrhagic syndromes throughout South America. *Arenaviridae* also includes lymphocytic choriomeningitis virus (LCMV), a well-studied virus that has facilitated many advances in the fields of virology and immunology, although not a major focus of this review [132].

Beyond studies involving LCMV, T cell responses against LASV and other mammarenaviruses have also been characterized [58,59,60,133]. These studies focused on identifying immunogenic epitopes against entire proteomes for several mammarenavirus strains (LASV, LUJV, CHAPV, JUNV, MACV, GTOV, and SABV), with the goal of identifying conserved epitopes among the family [58,133,134,135]. Immunoinformatic analysis identified several highly immunogenic epitopes, mostly all located in conserved regions of GPC and N [58]. Ex vivo stimulation of LF survivor cells narrowed down the panel of immunodominant epitopes to 12 CD8+ T cell-positive epitopes within GPC and N which induced broad peptide-specific T cell responses, supported by predictive HLA-binding algorithms (Table 1) [59,60]. Further, four immunodominant CD4+ T cell epitopes, which are highly conserved between Old and New World arenaviruses, were identified and mainly localized to a short stretch of 13 amino acids located in the N-terminal part of GP2 _(289–301)_ (Table 1) [61]. Another study also showed strong human memory CD4+ T cell responses against N during LASV infection [62]. In mice, CD4+ T cells specific to the GPC _(403–417)_ of LASV can mediate a cross-protective immunity to LCMV infection [63]. Notably, the immunogenicity of GPC peptide candidates was evaluated in HLA-A*0201 mice, which were protected against challenge with a recombinant vaccinia virus that expressed the LASV GPC [136,137].

In human LASV infection, T cells play a major role in controlling acute infection, as patients recover in the absence of a measurable nAb response [138,139] Furthermore, treatment with immune plasma did not protect LF patients, strongly suggesting a critical role of cell-mediated immunity against LASV infection in humans [140]. Survival and LASV clearance in humans correlate with robust virus-specific CD4+ and CD8+ T cell responses during acute stages, coupled with elevated early IFN levels [59]. In contrast, severe LF cases are associated with weak LASV-specific T cell responses and non-specific T cell activation [141,142]. Currently, our understanding of CD4+ T cell response to LASV infection is limited to observations of LASV-specific CD4+ T cells in convalescent patients [61,62].

Our knowledge about JUNV-specific T cell responses is restricted to a few mouse studies, which implicated T cells in the clearance of virus from infected organs and their correlation with disease severity [143,144]. The precise roles of CD4+ and CD8+ T cells, along with their epitope targets, remain unknown. Further investigations will help improve our understanding of the immunopathogenesis of JUNV and other arenavirus infections. Given that T cells play a protective role during infections with arenavirus, even in the absence of nAb responses, cross-protective T vaccines should be a major focus of future vaccine design and testing.

## 4. Antibody Responses against *Bunyavirales*

### 4.1. Peribunyaviridea

A distinctive characteristic of orthobunyaviruses is the genetic relatedness of viruses within serogroups, leading to cross-reactive Abs across the genus, including CSG and SSG members [145,146,147]. However, whether cross-reactive Abs can protect against multiple infections remains uncertain. Human and animal infections with orthobunyaviruses elicit nAbs, as evidenced by studies analyzing serum Abs against viral cell lysates with confirmatory neutralization assays [148,149,150,151]. In a separate study, individuals previously infected with INKV had strong Ab responses against N during the acute febrile phase, with more pronounced Gc Abs during convalescence [152]. These studies are noteworthy as they contribute to the limited research exploring human Ab responses against orthobunyaviruses, emphasizing a substantial gap in our understanding of the humoral response to these viruses.

Nevertheless, animal models have helped play a role in identifying specific proteins targeted by nAbs, revealing that envelope glycoproteins and N are the primary targets (Figure 1C). Mouse-derived monoclonal antibodies (mAbs) against LACV, TAHV, and SBV envelope glycoproteins and N were shown to be both specific and cross-reactive, but only the glycoprotein mAbs had neutralizing effects [153,154]. These findings were supported by several other studies on CSG and SSG serogroups, demonstrating the effect of nAbs against Gc [146,155,156]. However, the complex arrangement of envelope glycoproteins on the orthobunyavirus virion, characterized by trimeric spikes, has posed challenges in determining precise nAb epitopes [157]. The Gc protein, particularly the head domain (amino terminal subdomain), is targeted by LACV and SBV nAbs [158,159]. An X-ray crystallography study of the SBV glycoprotein also confirmed that mAbs bind to the projecting spikes, and that the immunization of mice with the head-stalk of Gc elicits sterilizing immunity [157]. Similar observations were reported for LACV and AKAV using mAbs produced in BALB/c mice [159,160,161]. High N-specific Ab titers are also frequently reported during infection with orthobunyaviruses. However, antibodies against N exhibit sub-neutralizing or non-neutralizing activity, as observed in mice and rabbits infected with LACV, TAHV, SBV, and the Cache valley virus (CCV) [147,153,154,155,162].

To evaluate the potency and efficacy of antigen-specific Ab responses to orthobunyaviruses, vaccine studies in animals have been crucial to improving our understanding [163,164,165]. IFNAR^−/−^ mice vaccinated with DNA encoding LACV Gc produced nAbs that exhibited a high degree of protection against LACV challenge [165,166]. In rhesus monkeys, a recombinant chimeric LACV expressing JCV surface glycoproteins induced cross-reactive nAbs against JCV, LACV, and TAHV, protecting against viremia after JCV infection [164]. BALB/c mice immunized with chimeric vesicular stomatitis virus encoding the OROV GPC demonstrated an nAb response, associated with reduced OROV viremia [167]. Additionally, IFNAR^−/−^ mice immunized with the SBV subunit of the Gc head domain were protected upon SBV challenge [157].

Numerous uncertainties surround Ab responses to orthobunyaviruses, necessitating further investigations. A critical aspect is the detailed mapping of human Abs to specific viral antigens, urging us to transcend the assumption that only structural glycoproteins and N are targeted. For example, Abs against dengue virus nonstructural protein 1 (NS1) proved to have protective effects in both mice and humans [168,169,170]. It will be important to study the involvement of non-structural proteins in eliciting Abs against orthobunyaviruses. Moreover, there is a need to analyze both neutralizing and non-neutralizing effector functions against these targets, probing whether they correlate with protection from severe disease. This comprehensive approach will deepen our understanding of the intricate dynamics of Ab responses to orthobunyaviruses that may aid in the development of both vaccines and Ab-based therapeutics.

### 4.2. Phenuiviridae

Studies on Abs isolated from human patients infected with RVFV, SFTSV, the Heartland virus (HRTV), and Guertu virus (GTV) have highlighted Gn as the primary target of nAbs, followed by N and Gc, which exhibit comparatively lower neutralizing activity (Figure 1C) [171,172,173,174]. Mapping the antigenic sites on RVFV envelope glycoproteins using mAbs has helped identify specific epitopes crucial for neutralization [175]. The crystal structures of RVFV and SFTSV glycoproteins help elucidate the mechanisms of neutralization [176]. The Gn structure of these viruses reveals three subdomains (domains I, II, and III), displaying a compact triangular shape [176]. Importantly, helices α6 in subdomain III of the Gn head are a key component for neutralization, as demonstrated by the structure of SFTSV Gn and human monoclonal nAbs [176]. The structural insights suggest that nAbs may impede phenuivirus glycoprotein rearrangement, hindering the exposure of fusion loops in Gc to endosomal membranes upon virus entry into the host cell [172,176,177]. The structure indicates that domain III is an ideal region recognized by specific nAbs, while domain II is likely recognized by nAbs that cross-react with related viruses [176]. Another study identified two major neutralization sites on RVFV Gn corresponding to positions (_173_TQEDATCK_180_) and (_271_CPPK_274_) [172]. Similar findings were observed using SFTSV human mAbs, binding a linear epitope in the ectodomain of Gn and effectively neutralizing all clinical isolates of SFTSV [178]. Additionally, a recent study identified two TOSV epitopes within the amino-terminal half of Gn as the primary targets for human nAbs [179]. In RVFV and TOSV infection, Abs targeting NSs have been reported, albeit in low levels [180,181,182].

Human infections with RVFV, SFTSV, and TOSV lead to the development of nAbs, exhibiting similar serological kinetics across all three viruses [171,178,183,184]. Patients infected with these viruses generate virus-specific IgM early at symptoms onset, with IgG Abs emerging around 15–30 days from onset, which can persist for years in convalescent sera [180,181,185,186]. Ab responses have been proven to contribute to protection and improved clinical outcomes in SFTSV-infected human patients. nAbs targeting SFTSV Gn play an essential role in the survival of patients with SFTS, detected in survivors but not fatal cases, potentially due to B cell class switching failure [187,188,189]. Several animal studies have also confirmed protective immunity post-RVFV infection and glycoprotein subunit vaccination, correlating with the development of virus-specific nAbs [172,190,191,192,193]. Intriguingly, the passive transfer of non-neutralizing Gn Abs demonstrated a capacity to restrict RVFV disease progression in BALB/c mice [194]. The efficacy of Abs against Gn SFTSV was also demonstrated in mice immunized with a Gn mRNA vaccine, producing robust nAbs that fully protect mice from a lethal dose of SFTSV, resulting in no fatalities [195]. Additional passive serum transfer experiments revealed that sera collected from IFNAR^−/−^ mice inoculated with recombinant SFTSV GPC, but not with N, conferred protective immunity against lethal SFTSV challenge in naïve mice [79].

Clearly, Ab responses are effective in protecting against infections with *Phenuiviridae* viruses, underscoring the importance of developing mAb-based therapeutics. The well-characterized nature of RVFV, SFTSV, and TOSV facilitates the design of Ab therapeutic strategies targeting broadly recognized antigenic epitopes, which could serve to protect against potential pathogenic viruses yet to emerge from this family.

### 4.3. Hantaviridae

Studies have consistently demonstrated that patient-derived Abs predominantly target Gn and Gc, followed by N (Figure 1C) [196,197]. Despite decades of research on hantaviruses, the intricate arrangement of Gn/Gc remains largely unknown, although four Gn protomers and four Gc protomers are thought to make up the surface exposed spikes [198,199]. It is noteworthy that Gn constitutes the distal part of the spike and is exposed to the extracellular space, in contrast to Gc, which is less exposed [200]. Recent antigenic mapping studies and the functional characterization of nAbs against hantaviruses have provided insights into their targets and mechanisms. Two broadly nAbs to SNV target the interface between Gn/Gc and domain I of Gc, neutralizing through fusion inhibition [201]. Another study characterized a highly potent SNV nAb targeting the Gn subcomponent of the heterodimer assembly, crucial for viral entry [202]. Other Abs specific to ANDV block viral entry, targeting different antigenic sites on the head domain of Gn [201]. Earlier studies mapped critical residues on Gc essential for neutralization against PUUV [203,204]. nAbs against PUUV Gc recognize conserved regions in the fusion loop sequences and the main chain of variable Gn sequences, effectively locking the Gn/Gc heterodimer in its prefusion conformation [205,206]. In contrast, non-neutralizing Abs against Gn, isolated in rabbits immunized with HTNV Gn, target spatially distinct epitopes in the N-terminal region of the HTNV Gn ectodomain [207]. Although less frequent, studies on Abs against N elicited during HTNV natural infection indicate the presence of N-specific IgG, particularly in early infection in human patients [196]. B cell epitopes in the PUUV N protein, evaluated in immunized bank voles, localized within the amino-terminal region of the protein, elicit N-specific IgG during early infection in human patients [116,208]. The in silico prediction of B cell epitopes in ANDV and SNV N proteins reveals promiscuous epitopes identified in the C-terminus of the protein [209].

Efforts to establish a link between Ab responses and protection against infection with hantaviruses have shown promising results. The preclinical evaluation of mAbs against Gn/Gc showed they were highly protective against lethal challenge in a Syrian hamster model of ANDV infection [210,211]. Single doses of an nAb recognizing both Gn and Gc protected Syrian hamsters and bank voles challenged with highly virulent ANDV and PUUV [206]. Early evidence indicates that an nAb response to either Gn or Gc alone is also sufficient to prevent HTNV infection in hamsters [212]. Furthermore, HTNV mAbs targeting glycoproteins have provided a protection against challenge in various rodent models [213,214]. Among a panel of murine mAbs recognizing HTNV N and Gn, only Gn-specific Abs provided full protection in vivo against HTNV infection in susceptible mice that received monoclonal nAbs one day before and two days after being exposed to HTNV [215].

In humans, the humoral response plays a significant role in providing protective immunity against hantaviruses [97]. Passive transfer of hyperimmune ANDV human sera to treat HCPS showed a decrease in the case fatality rate [216]. Low titers of IgG Abs are associated with moderate-to-severe disease outcomes of HFRS and HCPS [217,218,219,220,221,222,223]. Neutralizing mAbs isolated from SNV- and ANDV-infected human patients have shown therapeutic efficacy at clinically relevant doses in hamsters infected with these viruses [217].

Ab therapeutic trials for hantaviruses are primarily focused on targeting Gn and Gc, with the goal to generate robust and long-lasting nAbs responses [224]. Given the pivotal role of the humoral response in protection against multiple hantaviruses, future work should prioritize the development of broadly nAb therapeutics.

### 4.4. Nairoviridea

Despite limited structural information regarding the CCHFV envelope, Gc has been identified as the primary target of host nAbs (Figure 1C). Most mAbs used in antigen mapping are isolated from immunized mice [225,226]. A recent study addressed this gap by designing a trimeric protein including most of the ectodomain region of the CCHFV Gc [227]. The structure confirmed that CCHFV Gc is a class II fusion protein; unexpectedly, however, CCHFV Gc adopted hybrid architectural features of the fusion loops compared to hantaviruses and domain III from phenuiviruses [227]. The modeled target sites were validated by a separate study analyzing serum Abs from CCHFV human survivors [227,228]. These studies revealed six distinct sites in the Gc subunit targeted by potent Abs, with major neutralizing activity concentrated against the highly conserved fusion loop in the C-terminus of the Gc and domain II [228]. Abs targeting the fusion loop site effectively block the insertion of the fusion loop into the target membrane, while those binding to domain II prevent the conformational transition of Gc by blocking the formation of the post-fusion homotrimer [227]. Additionally, CCHFV encodes a secreted glycoprotein (GP38) of unknown function that is also a target of non-neutralizing Abs [229]. mAbs recognizing N were also reported in sera from humans infected with CCHFV and animals infected with NSDV [230,231,232].

Contrary to the direct correlation between neutralization and protective potency observed in some viral infections, the Ab response to CCHFV does not strictly adhere to this pattern. Studies testing nAbs specific to Gc in mice demonstrated partial or limited protection [225,233]. In contrast, non-neutralizing Abs targeting the Gn polyprotein precursor (pre-Gn) and/or GP38 provided protection in mice, especially when administered prior to viral challenge [225,233,234]. To date, 13G8 has been identified as the sole protective mAb against CCFHV in STAT1^−/−^ mice, demonstrating its binding to GP38 at a subnanomolar affinity [229]. Interestingly, the effectiveness of GP38-targeting Abs in providing protection depend on complement activity, suggesting that Ab effector functions, such as complement-mediated lysis and phagocytosis, play a crucial role in protecting against severe disease [233]. These findings underscore the potential utility of existing recombinant mAbs against CCHFV, while indicating the need for new mAbs with enhanced potency and additional functions beyond neutralization. Responding to this need, a recent study illustrated the efficacy of bispecific antibodies (bsAbs) by incorporating variable domains from a wide range of nAbs to boost their antiviral efficacy. The structural basis of the mechanism of action of these bsAbs shows the two Fabs (ADI-36121 and ADI-37801) acting in concert to block membrane fusion, with one targeting the fusion loops and the other blocking Gc trimer formation [235,236].

CCHFV infection triggers the production of nAbs in human patients, detected as early as 10 days after disease onset [237]. Notably, undetectable levels of nAbs are observed in fatal cases, while survivors exhibit low levels, suggesting that Abs may play a role in protection from lethal CCHFV infection [237]. Our knowledge about the efficacy of human Abs generated in response to CCHFV infection is limited to IgM and IgG seroprevalence studies, although a few recent studies isolated CCHFV-specific mAbs against glycoprotein and GP38, validating their protective efficacy in mice [229,235]. Nevertheless, a major gap in our knowledge regarding CCHFV is the unknown mechanisms of viral entry into the cell. As such, studies focusing on mAbs that can block viral entry, as well as target the GP38, of nairoviruses may help improve the design of future Ab therapeutics.

### 4.5. Arenaviridae

Distinct patterns of antibody responses are observed in Old and New World arenaviruses, reflecting differences in Abs and their protective potency [238,239]. While New World viruses typically elicit robust nAb responses, Old World viruses generally evade such responses (Figure 1C) [239]. However, in both cases, Abs primarily target the surface GPC [240]. Arenavirus GPC is composed of a receptor-binding subunit GP1 and a transmembrane fusion subunit GP2 [200].

The weak Ab response against LASV can be attributed to the unique structure of the LASV GPC, which mediates entry into target cells and is the primary target of nAbs [241,242]. The virion form of GPC is metastable and heavily glycosylated, presenting a thick carbohydrate coat that challenges the elicitation of nAbs [239,241]. The glycan shield mainly serves to evade immune responses and can undermine the protective, neutralizing capacity of Ab immunity [241]. Another challenge for the development of potent Abs is the existence of several distinct LASV lineages, each improving resistance to Ab neutralization [241,243].

Targets for nAbs in LASV survivors were identified in one of the largest anti-LASV Abs isolation studies to date, defining the canonical Abs competition groups: GP1-A, GPC-A, GPC-B, and GPC-C [244]. Half of the mAbs isolated bind the GP2 fusion subunit (GPC-B), one-fourth recognize the GP1 receptor-binding subunit (GP1-A), and the remaining fourth are specific to the assembled GPC, requiring both GP1 and GP2 subunits for recognition (GPC-C, GPC-A) [245,246,247,248]. nAbs recognize the same pattern of epitopes on the JUNV glycoprotein [249]. The isolated mAbs against GPC in mice and JUNV survivors strongly bind GP1, responsible for receptor recognition, mimicking an important receptor contact [249,250]. A JUNV GP2-directed mAb prevents membrane fusion by binding to an intermediate form of the protein on the fusion pathway [251]. mAbs specific to MACV were also found to have a potent neutralization activity in vitro against pseudotype and native MACV [252].

Protective Ab responses directed against viral proteins in animals have also been characterized. LASV GPC immunization using various formulations induces potent protective humoral responses in animals, also confirmed by passive transfer experiments [253]. Even though these immunization strategies mostly induce binding but non-neutralizing Abs, they still provide protection, likely facilitated by cellular immune responses or antibody-dependent cellular cytotoxicity [253,254,255]. LCMV induces Abs against N and GP2 soon after infection, reaching higher titers, whereas nAbs exclusively target GP1 and remain undetectable for the first two months after infection in mice [256,257,258,259].

nAbs may not be the sole determinate of survival in humans acutely infected by LASV. Surprisingly, half of individuals who successfully recover from LASV infection either fail to produce nAbs or do not achieve effective titers, even during late convalescence and several months of follow-up [140,260,261,262]. The development of low nAb titers may not occur until at least two months post-infection, a delay reflected in the persistence of IgM Abs against LASV GPC and a disruption in the expected class switching to IgG during the course of human infection [263].

Early attempts at passively transferred serum therapy in humans underscored the limited protective potency of nAbs against LASV infection [140]. The failure of this approach was directly linked to the use of whole plasma, which may contain low nAb levels, or the inability of Abs to target mutated virus strains [140]. Nevertheless, ongoing research aims to investigate whether nAbs with virus strain specificity, delivered in sufficient quantity, can serve as an effective treatment for LF when provided passively. The limited successful treatment of LASV infection in cynomolgus macaques and LASV patients has been reported, using plasma from LASV survivors as a treatment modality [140,262]. Conversely, administering neutralizing mAbs to non-human primates provided protection against severe LF, even when given at low doses and late in the disease course [264].

In contrast to LASV, nAbs play a crucial role in virus clearance for Argentine hemorrhagic fever (AFV) patients infected with JUNV [265]. Convalescent plasma stands out as the most promising, and currently the only, approved treatment for AHF [266]. Patients with AHF who were treated with immune plasma within eight days of disease onset had a much lower mortality rate than those given normal plasma [267]. Moreover, the generation of nAbs has been established as a key measure of successful vaccination against JUNV [268]. A potential therapeutic approach, utilizing a humanized anti-GPC neutralizing mAb, demonstrated protective efficacy against JUNV challenge in non-human primates [269]. Notably, while a variety of potential vaccines have been explored, the only available vaccine for an arenavirus is the live-attenuated Candid#1 strain of the Junin virus, which is exclusively licensed in Argentina and has been in use since 1992 for people at risk. The FDA has not approved this vaccine due to the possibility that the virus may revert back to a more transmissive or pathogenic strain [270].

Considering the promising prospects of mAb therapy for arenaviruses, future research should prioritize enhancing the neutralizing potency of Abs for more efficient and potentially cross-functional therapeutic use. Moreover, relying solely on plasma transfer as a therapy for JUNV is challenging due to limitation in quantity, variability in quality, and inherent safety risks, such as the potential transmission of transfusion-borne diseases. A deeper characterization of human mAbs is essential to improve the array of therapy options available for LASV, JUNV, and other arenaviruses.

## 5. *Bunyavirales* Vaccines and Therapeutic Strategies

The history of *Bunyavirales* vaccines is marked by the absence of licensed or globally approved vaccines for human use against any bunyavirus. In response to RVFV outbreaks, two single-dose live-attenuated vaccines (DDVas and RVFV-4S) are undergoing preclinical development for potential human use [271]. Another promising candidate, an adenovirus-vectored vaccine (ChAd-Ox1 RVF) expressing RVFV glycoproteins, has advanced to phase I clinical studies following demonstrated efficacy in animal models [272,273]. Additionally, the inactivated virus vaccine Hantavax, targeting HTNV and the Seoul virus (SEOV), has progressed to human clinical trials in Korea and China [274,275]. Encouragingly, ongoing phase 2 trials in the US are evaluating DNA-based vaccines targeting HTNV, PUUV, and ANDV [224].

The slow progress in developing effective *Bunyavirales* vaccines can be attributed in large part to the lack in research funding and the unclear guidelines for producing vaccine candidates against these relatively newly emerging and diverse viruses. Despite considerable efforts to assess vaccine efficiency in animal models, the preference between inducing T cell responses or Abs remains unclear. Furthermore, safety and efficacy elements have not been adequately explored for *Bunyavirales* vaccination trials in animals. In certain cases, such as orthobunyaviruses, the lack of knowledge concerning the host adaptive immune response impedes the prediction of vaccine candidate behavior.

**Figure 2:**
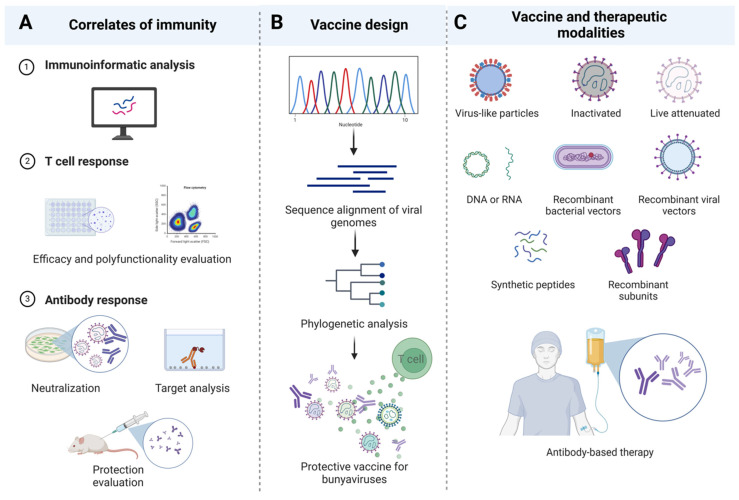
Vaccine design informed by studies on mechanistic correlates of immunity. (**A**) Vaccine design necessitates in-depth characterization of mechanistic correlates of immunity involving immunoinformatics analysis to help identify T cell and antibody targets, followed by ex vivo and in vivo characterization of adaptive immune responses during infection and/or vaccination. (**B**) Design of vaccine candidates should be tailored towards preventing or treating infections by specific viruses, with a long-term goal of potentially developing and testing cross-protective *Bunyavirales* vaccines. (**C**) Various delivery modalities can be employed for vaccine candidates including traditional approaches like inactivated/live attenuated viruses or protein-based vaccines, or more novel methods such as mRNA vaccines or monoclonal antibody therapies.

*Bunyavirales* vaccines should ideally generate a balance of potent T cells and nAbs capable of clearing the virus. This is made possible by an in-depth characterization of the mechanistic correlates of immunity during infection, disease, and/or vaccination (Figure 2A). However, defined correlates of immunity have yet to emerge for most viruses within the *Bunyavirales* order. For example, glycoprotein-based vaccines in the *Phenuiviridae* family, such as DNA vaccination against SFTSV glycoprotein, mainly produce cell-mediated immunity with no detectable Abs against the glycoprotein [76]. In contrast, glycoprotein-based vaccines against RVFV induce strong protective nAbs [172,190,193]. Notably, non-neutralizing glycoprotein Abs can also restrict RVFV disease progression in mice [194]. In the *Hantaviridae* family, evidence from glycoprotein recombinant vaccines shows that Abs alone are sufficient to protect against infection, while glycoprotein peptide-based vaccines in other studies induce strong CD8+ T cell responses [212]. For the *Nairoviridae* family, CCHFV glycoprotein vaccines primarily promote protection through CD8+ T cell-,mediated mechanisms, with neutralization not proven necessary for protection, as GP38 vaccines achieve protection though non-neutralizing Abs [122,123,233]. Similarly, glycoprotein-based vaccines provide protection primarily via cellular immunity against LASV infection [254,255]. Furthermore, vaccines targeting the N protein have faced challenges in inducing full protection for certain bunyaviruses in animals, while N mRNA vaccines induced protection against CCHFV infection mostly through cell-mediated responses [35,79,123]. Non-structural proteins have not been considered as vaccine candidates for any bunyaviruses, although their efficacy remains to be determined.

The induction of potent nAb responses seems to be preferential for viruses within *Phenuiviridea*, *Hantaviridea*, *Nairoviridae*, and *Arenaviridae* (New World viruses). Given the potency of Abs against these viruses, Ab-based therapies have been considered as a post-exposure treatment modality. mAbs have been identified in animals to protect against some bunyavirus infections, such as anti-GP38 in CCHFV [233], anti-glycoprotein in LASV [264], and anti-glycoprotein JUNV [276]. A patent for the humanized Ab against SFTSV has also recently been registered (CN102942629B) [178]. Studies involving neutralization assays and the passive transfer of serum from immunized or infected animals to recipients provide insights into potential Ab-based therapeutic options [212,221,277,278]. An understanding of adaptive immune responses has also guided the exploration of treatment options based on cytokine mediators. For instance, the transfer of Abs to block specific cytokines, as in the case of SFTSV, is thought to provide protection [279]. Notably, the transfer of anti-IL-6 Abs significantly increased the survival of mice following SFTSV infection [279]. This approach is particularly relevant, given that SFTSV infection induces the production of high levels of IFN-γ and IL-6 in the serum, lymph nodes, and spleen [279].

Despite these developments, innovative vaccine approaches capable of inducing potent T cell responses have been explored in certain infections, where T cells are implicated as a crucial correlate of protection [280,281]. This is particularly noteworthy in CCHFV and LASV, as well as being potentially applicable to all other viruses within the *Bunyavirales* order [122,270]. For example, non-infectious bacterial toxins have been shown to deliver full-length viral antigens into the cells to induce potent CD4+ and CD8+ T cell responses via the MHC I and II pathways [282,283,284,285]. Whether these fusion immunogens can serve as T cell-based vaccines to help improve Ab-based vaccines and therapeutics in the context of *Bunyavirales* infections is an area of active investigation.

Creating an ideal *Bunyavirales* vaccine is a complex process that involves various other considerations. *Bunyavirales* outbreaks are infrequent; however, when they do occur, they do so most often in resource-limited regions [286,287,288,289]. This phenomenon has resulted in a lack of emphasis on bridging the gaps necessary to develop vaccines against these viruses. With constrained support, the comprehensive characterization of adaptive immune responses to each specific virus becomes an impractical endeavor. Consequently, the most viable option to developing effective *Bunyavirales* vaccines likely relies on the design and testing of universal, cross-protective vaccines capable of targeting multiple bunyaviruses within each of the families (Figure 2B). The observed cross-reactivity within each viral family lends feasibility and applicability to such an approach, opening avenues for significant advancements in combating these infectious threats, regardless of vaccine or therapeutic modality (Figure 2C) [145,173,290,291,292,293].

## 6. Concluding Remarks

With no globally approved vaccines for any virus in the order and only a few in early stages of clinical trials, the current state of *Bunyavirales* awareness requires strategic interventions. Despite the efforts addressed in this review to understand the dynamics of virus-specific adaptive immune response, substantial gaps persist in the field, emphasizing a need for strategies to address the challenges in vaccine development and the study of diseases induced by viruses within the *Bunyavirales* order.

## Figures and Tables

**Table 1:** *Bunyavirales* T cell epitopes predicted by immunoinformatic analysis and/or confirmed by ex vivo and in vivo studies.

Virus	T Cell Type	Epitope	Host	Approach
*Peribunyaviridae*				
OROV	CD8+	Glycoproteins TSSWGCEEY _(1043–1051)_ CSMCGLIHY _(48–56)_ LAIDTGCLY _(4–12)_	Humans	Immunoinformatics [30]
BUNV	CD8+/CD4+	N protein KRSEWEVTL _(55–63)_ AIGIYKVQRKEMEPK _(161–75)_	Humans	Immunoinformatics [32]
		Glycoproteins YQPTELTRS _(716–724)_ YKAHDKEET _(782–790)_ ILGTGTPKF _(1172–1180)_		Immunoinformatics [33]
JCV	CD8+	N protein AAKAKAALA _(26–34)_ AALARKPER _(152–161)_ ADHGESVSL _(175–183)_ ADHGESVSLS _(157–165)_ YPLTIGIYRV _(108–117)_	Humans	Immunoinformatics [31]
	CD4+	N protein AALARKPER _(146–154)_ ADHGESVSL _(160–169)_ DVEQLKWGR _(119–127)_ EIYLSFFPG _(183–191)_ FLIKFGVKL _(141–149)_		
SBV	CD8+	N protein Glycoprotein Gc _(678–947)_	IFNAR^−/−^ mice	Ubiquitinated and non-ubiquitinated cDNA immunization [34]
LACV	CD4+	Glycoprotein N protein	IFNAR^−/−^ mice	DNA vaccination [35]
SBV	CD8+	N protein	IFNAR^−/−^ mice	Bacterially expressed (SBV-N) [36]
*Phenuiviridae*				
TOSV	CD4+	N protein VKMMIVLNL _(58–66)_ Glycoprotein VMILGLLSS _(824–832)_	Humans	Immunoinformatics [37]
SFTSV	CD8+/CD4+	Panel of peptides 8 peptides within RdRp 8 peptides within glycoprotein	Humans	Immunoinformatics [38]
RVFV	CD4+	Glycoprotein LPALAVFALAPVVFA _(139–153)_ PALAVFALAPVVFAE _(140–154)_ GIAMTVLPALAVFAL _(133–147)_ GSWNFFDWFSGLMSW _(1138–1152)_ FFLLIYLGRTGLSKM _(1174–1188)_ N protein HMMHPSFAGMVDPSL _(143–158)_	Humans	Immunoinformatics [39]
	CD8+	Glycoprotein AVFALAPVV _(143–151)_ LAVFALAPV _(142–150)_ FALAPVVFA _(145–153)_ VFALAPVVF _(144–152)_ IAMTVLPAL _(134–142)_ FFDWFSGLM _(1142–1150)_ FLLIYLGRT _(1142–1150)_ N protein MMHPSFAGM _(144–152)_		
	CD8+/CD4+	Panel of peptides 14 peptides within N 13 peptides within Gn 16 peptides within Gc	Humans	Ex vivo stimulation assays/immunoinformatics [40]
	CD8+	N protein VLSEWLPVT _(121–129)_ ILDAHSLYL _(165–173)_	Humans	Ex vivo assays using N-transduced dendritic cells primed with CD8 T cells from HLA-A2 donors [41]
	CD8+	N protein NAAVNSNFI _(201–210)_	Mice C57BL/6	Ex vivo stimulation assay of vaccinated mice [42]
	CD4+	N protein VREFAYQGFDARRVI _(25–40)_ AYQGFDARRVIELLK _(29–44)_		
*Hantaviridae*				
Orthohantaviruses (multiple)	CD8+/CD4+	A panel of cross-reactive epitopes between multiple orthohantaviruses 6 peptides within glycoprotein 2 peptides within nucleocapsid 2 peptides within RdRp 1 peptide within NS protein	Humans	Immunoinformatics [43]
HTNV	CD8+	N protein NAHEGQLVI _(12–20)_ ISNQEPLKL _(421–429)_	Humans	Ex vivo stimulation [44]
		N protein TSFVVPILLKALYML _(127–141)_ YMLTTRGRQTTKDNK _(139–153)_ IEPCKLLPDTAAVSL _(241–255)_ LRKKSSFYQSYLRRT _(355–369)_	Humans	Ex vivo stimulation [45]
		N protein RYRTAVCGL _(197–205)_ KLLPDTAAV _(245–253)_ GPATNRDYL _(258–266)_	Humans	Ex vivo stimulation [46]
HTNV/SNV	CD8+	ILQDMRNTI (HTNV, aa _334–342_; SNV, aa _333–341_)	Humans	In silico prediction of conserved epitopes, validation of peptides ex vivo using patients’ PBMCs [47]
	CD8+/CD4+	N protein ERIDDFLAA _(234–242)_ LPIILKALY _(131–139)_ GIQLDQKIII _(372–380)_	Humans	Ex vivo stimulation assays [48]
HTNV		Glycoprotein LIWTGMIDL _(358–366)_	Humans	In silico prediction and evaluation of efficacy in transgenic mice [49]
ANDV	CD8+	Glycoprotein Gn SLFSLMPDVAHSLAV _(461–475)_	Humans	Ex vivo stimulation [50]
PUUV	CD8+	Glycoprotein Gn HWMDATFNL _(731–739)_	Humans	Ex vivo stimulation [51]
*Nairoviridae*				
CCHFV	CD8+/CD4+	Panel of peptides 3 peptides within Gc 2 peptides within Gn	Humans	Immunoinformatics [52]
		A panel of peptides 5 peptides within N protein 4 peptides within Glycoprotein	Humans	Immunoinformatics [53]
		RdRp DCSSTPPDR _(197–202)_	Humans	Immunoinformatic [54]
	CD8+	A panel of peptides 4 peptides within NSm 5 peptides within GP38	Humans	Ex vivo stimulation [55]
		N protein Gc	Humans	Ex vivo stimulation [56]
		Gc NSm	C57BL/6 mice	Ex vivo stimulation [57]
*Arenaviridae*				
LASV	CD8+	Glycoprotein MRMAWGGSY _(192–200)_ N protein ALTDLGLIY _(201–210)_	Humans	Immunoinformatics [58]
	CD4+	A panel of peptides 4 peptides within glycoprotein 4 peptides within N protein	Humans	Immunoinformatics [58]
LASV	CD8+	A panel of peptides 12 epitopes within glycoprotein and/or N	Humans	Ex vivo stimulation [59]
		N _(1–91)_ N _(411–491)_ GP2 _(92–172)_	Humans	Ex vivo stimulation [60]
LASV	CD4+	4 highly conserved peptides between Old and New World arenaviruses within GP2 _(289–301)_	Humans	Ex vivo stimulation [61]
		6 peptides within N protein	Humans	Ex vivo stimulation [62]
LASV/cross-reacts with LCMV	CD4+	Glycoprotein IEQQADNMITEMLQK _(403–417)_	C3H/HeJ mice	Ex vivo stimulation [63]

## Data Availability

All data are available within the manuscript.
